# Small bowel lymphoma presenting as inguinal hernia: case report and literature review

**DOI:** 10.1186/s12957-018-1396-4

**Published:** 2018-05-15

**Authors:** Michele Teodoro, Maurizio Mannino, Marco Vitale, Edoardo Mattone, Valentina Palumbo, Filippo Fraggetta, Adriana Toro, Isidoro Di Carlo

**Affiliations:** 1 Department of Medical, Surgical Sciences and Advanced Technologies “G.F. Ingrassia,” Cannizzaro Hospital, University of Catania, Via Messina 829, 95126 Catania, Italy; 2 Pathology Department, Cannizzaro Hospital, Catania, Italy; 3 General Surgery, Patti Hospital, Patti, ME Italy

**Keywords:** Bowel, Lymphoma, Inguinal, Hernia

## Abstract

**Background:**

Inguinal hernia is one of the most common benign pathologies that primarily affects men. Primary gastrointestinal non-Hodgkin’s lymphoma (PGI NHL) is the most common type of extranodal lymphoma. This study reports a rare case in which these two conditions co-exist.

**Case presentation:**

An 85-year-old male complained of bowel movement pattern change, abdominal distension and loss of weight, without vomiting but with nausea. A computed tomographic scan of the abdomen showed a small bowel obstruction caused by a migration of a small bowel loop in the right inguinal canal, with a clinically non-reducible inguinal hernia. The patient underwent surgery. The histopathological report showed small bowel large B cell non-Hodgkin’s lymphoma.

**Conclusion:**

When the diagnosis of the contents of an inguinal hernia is not well-established, surgery should be performed as soon as possible to ensure the cure of the disease and the correct diagnosis of the contents.

## Background

Inguinal hernia is one of the most common benign pathologies that primarily affects men; surgical hernia repair procedures account for approximately 2 million surgical cases annually worldwide, with 850,000 of those performed in the USA [1].

The small bowel [2] is the second leading site, after the stomach [3], for primary gastrointestinal non-Hodgkin’s lymphoma (PGI NHL). It commonly causes abdominal pain, changes in bowel habits, weight loss, a palpable abdominal mass and blood in the stool [4]. The most common histological subtype of PGI NHL is diffuse large B cell lymphoma, for which systemic chemotherapy with rituximab is the most common treatment [3].

Here, we report a rare case in which these two conditions co-exist and their treatment.

## Case presentation

**Fig. 1:**
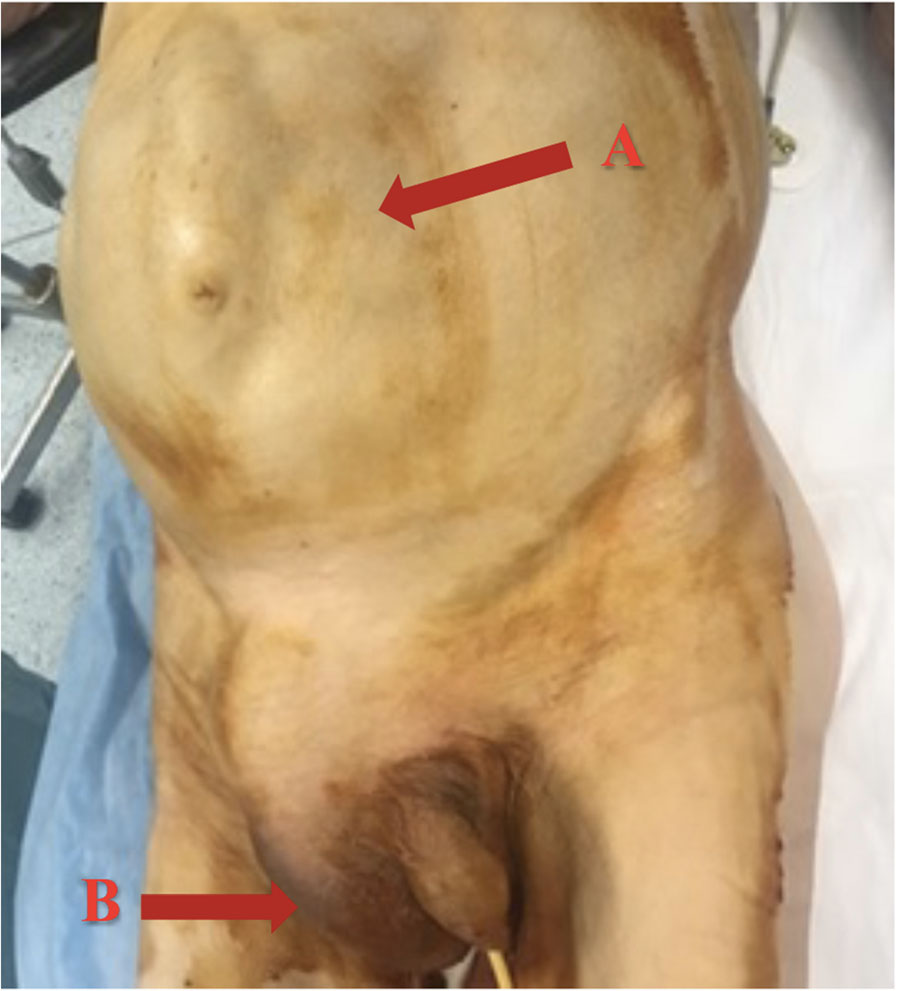
A: abdominal distension with a marked loop under the wall. B: giant scrotal mass located on the right side. Non-reducible

**Fig. 2:**
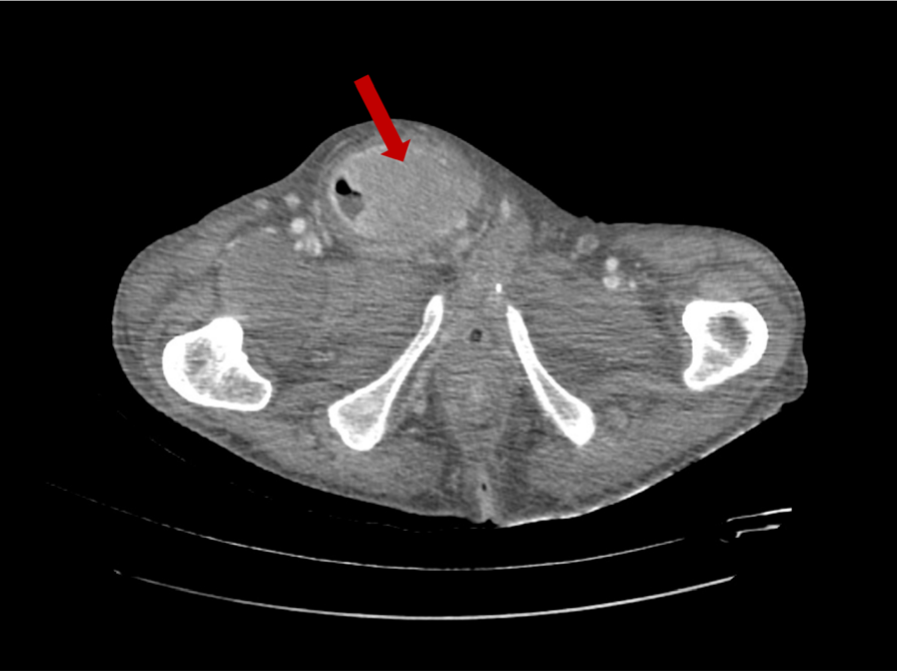
CT scan. The ileal loop located in the inguinal canal judged by the radiologist to be fixed and non-reducible

An 85-year-old male was admitted to the hospital complaining of an approximate 1 month of *bowel* movement pattern change, abdominal distension and loss of weight, without vomiting but with nausea. Clinical examination revealed abdominal distension (Fig. 1 A) and diffuse tenderness without signs of peritonitis. The patient had no lymphadenopathy, his pulse rate was 85 beats/min and his blood pressure was 105/60 mm/Hg. There was a large, painful and non-reducible swelling in the right inguinoscrotal region that was firm in consistency (Fig. 1 B). His total WBC count was 13.900/mm^3^, and his neutrophil count was 96.9%. A computed tomographic scan of the abdomen showed a small bowel obstruction caused by a migration of a small bowel loop in the right inguinal canal, with full-thickness expansion of the bowel wall (Fig. 2). The patient was taken to the operating room for surgery, and because the CT scan showed a mass firmly adherent to the inguinal canal, an abdominal approach was chosen for the possibility of an ileal resection.

**Fig. 3:**
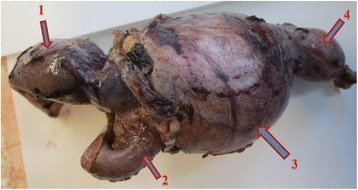
Specimen. 1: afferent loop, 2: efferent loop, 3: tumour mass, 4: testicle

**Fig. 4:**
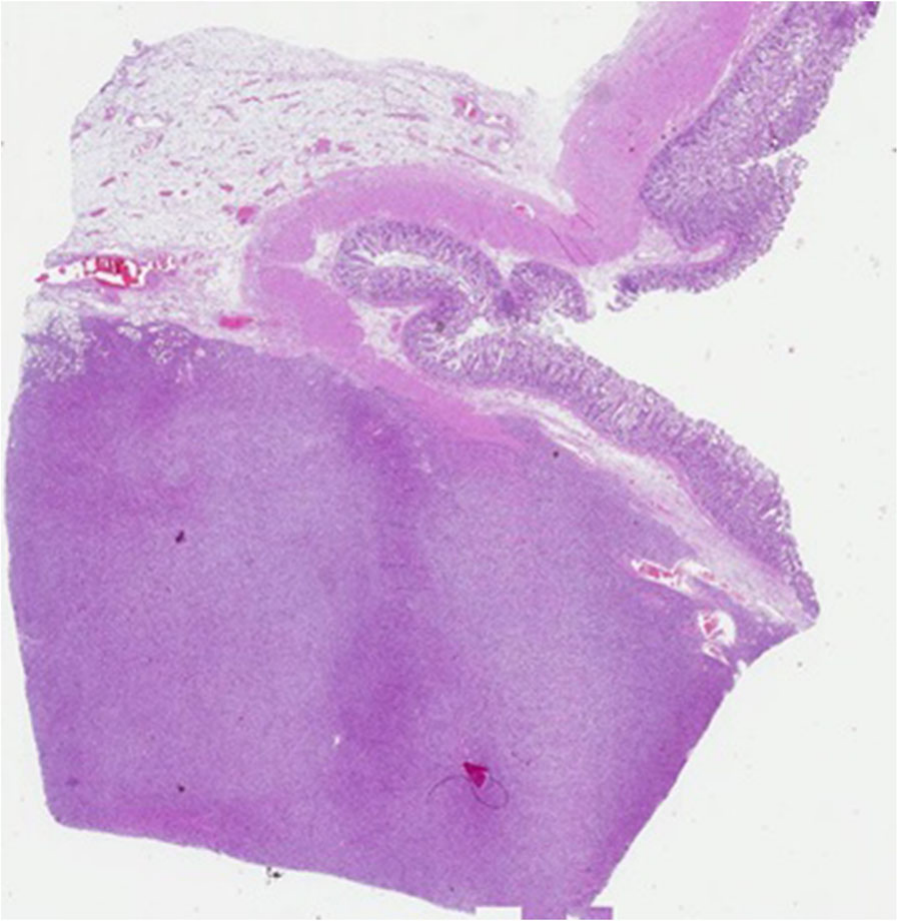
Microscopic view of the intestine. The mucosa is not affected (× 5)

**Fig. 5:**
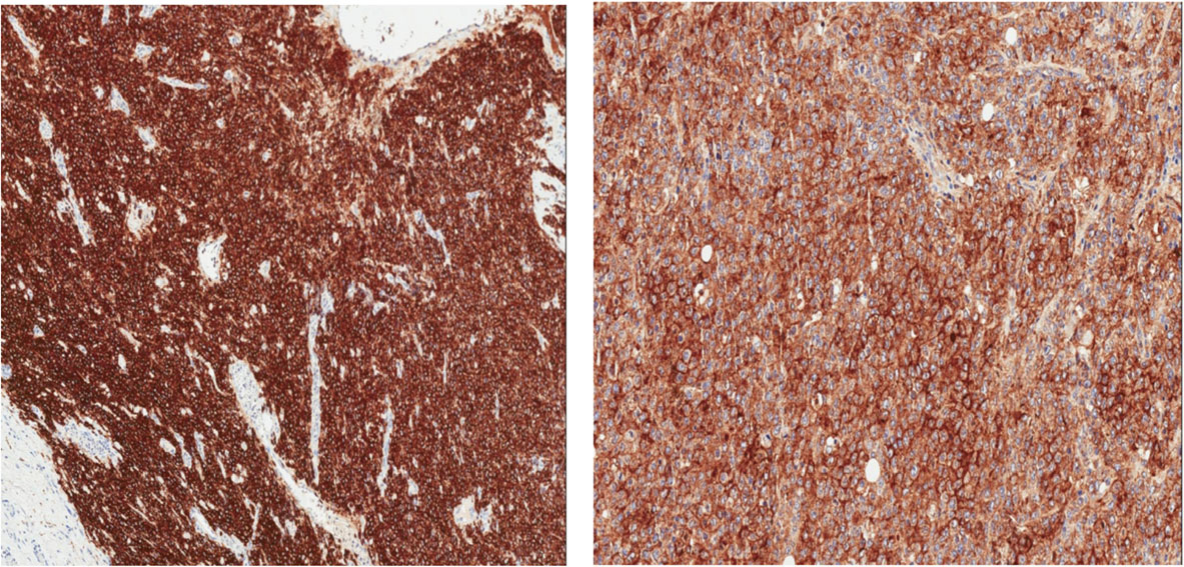
Positivity for CD30 and CE20 (× 10)

On exploration, fluid was found in the peritoneal cavity, and the ileal loop was lodged within the interior inguinal ring. A combined inguinal approach was deemed necessary. On exploration, a small bowel mass firmly adherent to and inseparable from the testicle was found. A bowel resection including the mass and the testicle was performed (Fig. 3). An ileo-ileal latero-lateral anastomosis was performed. Then, a Shouldice herniorrhaphy was performed. We decided to perform a herniorrhaphy without mesh use to avoid the risk of infectious complications. No complications in the post-operative period were recorded. The histopathological report showed small bowel large B cell non-Hodgkin’s lymphoma. The tumour was present up to the submucosa (Figs. 4 and 5). After discharge, the patient was referred to the Haematology Department for further treatment.

## Discussion and conclusion

Approximately, 10% of inguinal hernias become irreducible; this is often due to benign factors, whereas uncommonly, co-existing malignancy can be the cause of the irreducibility [5].

Malignant masses in inguinal hernias appear in less than 0.5% of excised sacs [6].

Tumours in the hernia sac can be classified based on Lejar’s studies as saccular and intrasaccular: saccular when they are primary, such as mesothelioma or when metastatic disease involves the peritoneal sac; intrasaccular when the sac contains an organ affected by a malignant tumour [6].

The majority of intrasaccular tumours are of intestinal origin, and the first case that was reported was a sigmoid colon carcinoma [7].

The sigmoid colon is involved in most cases; however, reported cases do not exceed a maximum of four cases per author. The most recent review reported a total of 27 colon carcinoma cases within inguinal hernia sacs and represents most of these patients present in the literature [8].

Other tumours of the intestine are rarely encountered, and they usually originate from the small bowel; eight such tumours have been reported to date [6, 9–15].

Other types of intrasaccular tumours, less frequent than the tumours of intestinal origin, include ovarian and bladder cancer tumours. Ovarian tumours presenting as an inguinal hernia have been described in ten cases, with patients ranging in age from 43 to 81 years [16]. Inguinal hernias containing a tumour of the urinary bladder are very rare, with only 22 cases reported in the literature [17].

Lymphoma diagnosed as an inguinal/femoral hernia is described in a few cases. A review of David R. and colleagues [18] reported 13 cases of lymphoma diagnosed as an inguinal or a femoral hernia, and all these cases originated from the nodes.

Only one case in the literature is reported as small intestine lymphoma diagnosed as a femoral hernia [19], and one case is reported of sigmoid colon lymphoma presenting as an irreducible left-sided inguinal hernia [4]; however, a review of the literature revealed no cases of small intestinal lymphoma presenting as an inguinal hernia (Table 1). This is the first reported case to the best of our knowledge. In our patient, the inguinal approach combined with an abdominal incision was mandatory due to the inability to reduce the hernia. This was probably due to the time lapse of anatomical defect. In fact, the presence of a tumour (in/to the herniated intestine), slowly but progressively increasing in size, does not permit the reduction of the herniated sac in the abdomen. Furthermore, this condition caused strong adhesions between the intestine loop and the sac and between the sac and the testicle. The main difference between this case and a routine case of an inguinal hernia was mainly the inability to manage the sac contents, resulting in a concomitant abdominal approach.

**Table 1:** Intrasaccular tumours of the inguinal canal, both carcinomas and lymphomas

Years	Authors	Site of the tumour	Number of cases reported
2008	Slater [8]	Colon	27
2003–2017	Different authors [6, 9–15]	Small bowel	8
2003	Takeuchi [16]	Ovary	10
2014	Katsourakis [17]	Bladder	22
2010	Veal [18]	Nodes lymphoma	13
2009	Assar [19]	Intestine lymphoma	1
2017	Di Carlo present case	Intestine lymphoma	1
		Total	82

In conclusion, this case report suggests that when the diagnosis of the contents of an inguinal hernia is not well established, surgery should be performed as soon as possible to ensure that the disease is cured and that the correct diagnosis of the contents is determined. In cases of a curable tumour, the lapse of time may be detrimental to the patient.

## Abbreviations

PGI NHL: Primary gastrointestinal non-Hodgkin’s lymphoma
WBC: White blood cell
